# *Alu* element in the RNA binding motif protein, X-linked 2 (*RBMX2*) gene found to be linked to bipolar disorder

**DOI:** 10.1371/journal.pone.0261170

**Published:** 2021-12-16

**Authors:** Pia Laine, William J. Rowell, Lars Paulin, Steve Kujawa, Denise Raterman, George Mayhew, Jennifer Wendt, Daniel L. Burgess, Timo Partonen, Tiina Paunio, Petri Auvinen, Jenny M. Ekholm

**Affiliations:** 1 Institute of Biotechnology, University of Helsinki, Helsinki, Finland; 2 Pacific Biosciences, Menlo Park, CA, United States of America; 3 Roche Sequencing Solutions, Madison, WI, United States of America; 4 Department of Public Health Solutions, National Institute for Health and Welfare, Helsinki, Finland; 5 Department of Psychiatry, University of Helsinki, Helsinki, Finland; Odense University Hospital, DENMARK

## Abstract

**Objective:**

We have used long-read single molecule, real-time (SMRT) sequencing to fully characterize a ~12Mb genomic region on chromosome Xq24-q27, significantly linked to bipolar disorder (BD) in an extended family from a genetic sub-isolate. This family segregates BD in at least four generations with 24 affected individuals.

**Methods:**

We selected 16 family members for targeted sequencing. The selected individuals either carried the disease haplotype, were non-carriers of the disease haplotype, or served as married-in controls. We designed hybrid capture probes enriching for 5-9Kb fragments spanning the entire 12Mb region that were then sequenced to screen for candidate structural variants (SVs) that could explain the increased risk for BD in this extended family.

**Results:**

Altogether, 201 variants were detected in the critically linked region. Although most of these represented common variants, three variants emerged that showed near-perfect segregation among all BD type I affected individuals. Two of the SVs were identified in or near genes belonging to the RNA Binding Motif Protein, X-Linked (*RBMX*) gene family—a 330bp *Alu* (subfamily *AluYa5)* deletion in intron 3 of the *RBMX2* gene and an intergenic 27bp tandem repeat deletion between the *RBMX* and G protein-coupled receptor 101 (*GPR101)* genes. The third SV was a 50bp tandem repeat insertion in intron 1 of the Coagulation Factor IX (*F9*) gene.

**Conclusions:**

Among the three genetically linked SVs, additional evidence supported the *Alu* element deletion in *RBMX2* as the leading candidate for contributing directly to the disease development of BD type I in this extended family.

## Introduction

Bipolar Disorder (BD) is a severe psychiatric disorder that is characterized by recurrent episodes of mania and depression. The lifetime prevalence is 2.4% [1] and the average age of onset is in the early twenties [1]. Based on twin studies the overall heritability for BD is 40–70%, and lifetime risk in first-degree relatives is 5–10% which is almost seven times higher than the general population risk [1].

Common complex disorders, such as bipolar disorder, are thought to be caused by a combination of genetic and environmental factors. To unravel the complex genetic makeup of these disorders, genome-wide association (GWAS) and family-based linkage studies have been applied. For BD, a number of significant linkage and association findings have been reported. One of these studies was by Pekkarinen et al. who reported significant linkage (lod score: 3.54) in a genome-wide survey to chromosome Xq24-q27 in a Finnish extended pedigree segregating BD in four generations [2]. A follow-up study based on microsatellite markers narrowed down the critically linked region to 12Mb [3]. Despite continued SNP mapping efforts, the critical region could not be further reduced, nor could a disease variant be pinpointed. This is not an uncommon phenomenon for common complex disease. To date, thousands of single nucleotide polymorphisms (SNPs) have been associated with various common complex disease phenotypes [4]; however, the effect sizes typically are small and explain only a portion of the disease heritability [5, 6]. Similar conclusions were drawn for the largest GWAS meta-analysis that was performed by the PGC Bipolar Disorder Working Group. They analyzed 20,353 cases and 31,358 controls, and identified 30 genome-wide significant loci, of which 20 were novel [7]. One emerging hypothesis holds that the missing heritability is hidden in other types of genetic variation than SNPs, such as structural variants (SVs) of >50 bp [8] in length, whose effects are not adequately represented by neighboring SNPs [5].

**Fig 1:**
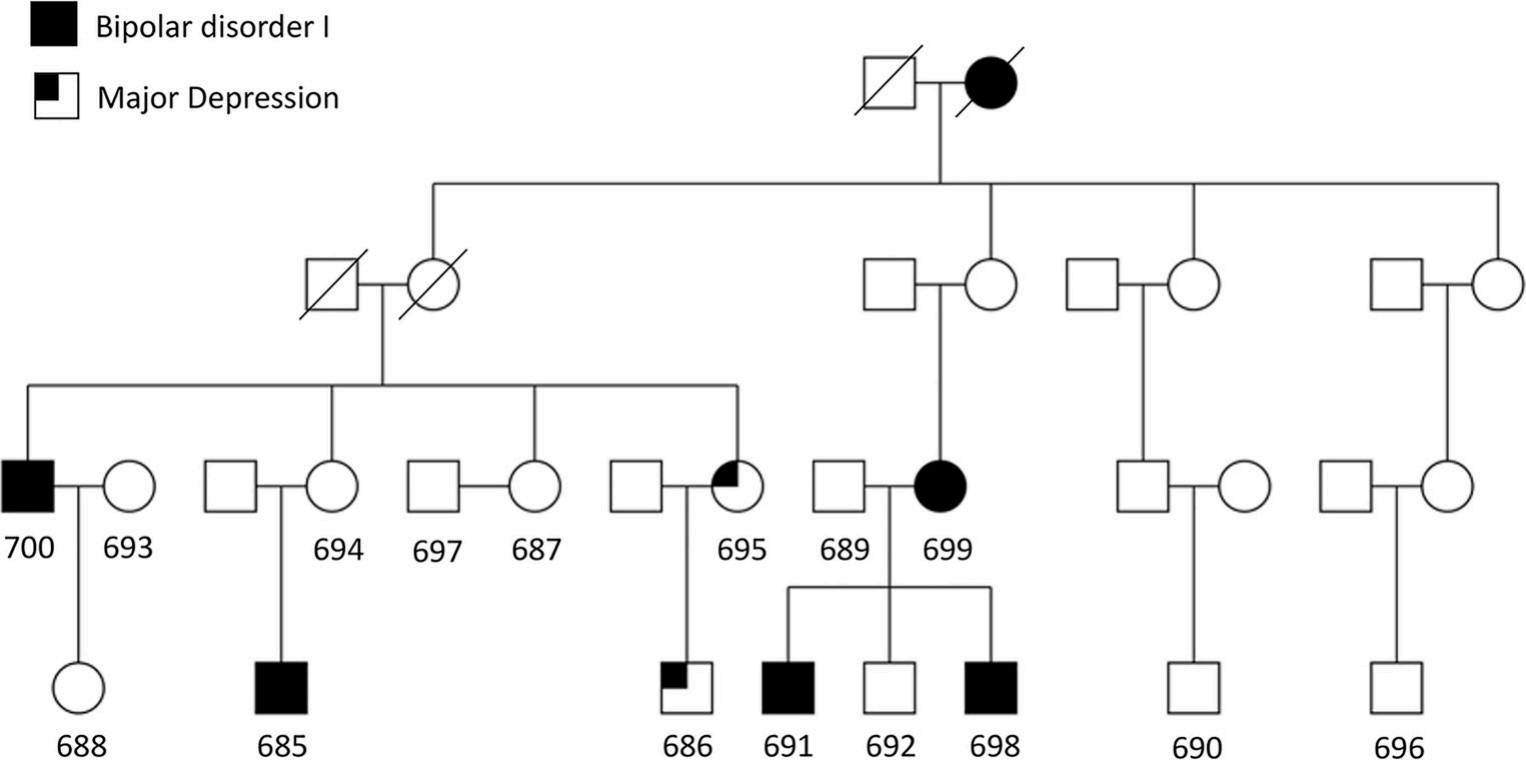
Extended family P101. Partial family P101 pedigree that displays the 16 individuals that were included in this study. These included five affected males (bipolar disorder I: 700, 685, 691, 698, major depression: 686) and two affected females (bipolar disorder I: 699, major depression: 695). In addition, three married-in unaffected controls were included (693, 697, 689) as well as six unaffected family members (688, 694, 687, 692, 690, 696).

In the human genome there are over 25,000 SVs and, despite not being as common as SNPs, due to their size they make up 60% of all variant bases in the human genome [9]. Genome-oriented studies have shown that SVs like insertions, deletions, duplications, translocations, inversions, and tandem repeat expansions can all cause disease [10–12] for example through the disruption of key gene regulatory elements [5]. Over the past several decades, the discovery of new SVs has followed closely behind technological advances in sequencing platforms. Therefore, we wanted to explore the possibility of a SV being the driver of the linkage signal to chromosome Xq24-q27 in the extended pedigree enriched with BD originally reported by Pekkarinen and colleagues [3]. Altogether, 24 out of 61 (39.3%) members in this family were diagnosed with some form of psychiatric disorder (Fig 1), therefore presenting a much higher life-time prevalence than the general population (2.4%). The hypothesis was that this extended pedigree would present a less heterogenous form of bipolar disorder because it stemmed from a genetic sub-isolate in Finland and showed no significant linkage to other genomic regions. To this effect, we used long-read sequencing to screen the 12Mb genomic region for a potential disease-causing SV in key individuals for the extended pedigree. Single Molecule, Real-Time (SMRT) long-read sequencing (Pacific Biosciences, CA, USA) has shown to discover 80% more SVs than other sequencing methods [13] through recent human genome assembly studies. In addition, long sequencing reads also help resolve structural breakpoints and to define allele-specific haplotypes [14].

In this study we detected altogether 201 SVs from 16 key individuals in the critically linked 12Mb genomic region. Although most of these represented common variants that could be seen across many of the family members regardless of disease status, or in the general population, three of the SVs showed near-perfect segregation among affected individuals that were identified as carriers of the disease haplotype in the previous linkage studies [2, 3]. Two of these were located within the same gene family–one SV was a 330bp antisense *Alu* deletion in intron 3 of the RNA Binding Motif Protein, X-Linked 2 (*RBMX2*) gene, while another variant, a 27bp tandem repeat deletion, was located in the intergenic region of the RNA Binding Motif Protein, X-Linked (*RBMX)* and G protein-coupled receptor 101 *(GPR101)* genes. The third SV was a 50bp tandem repeat insertion located in intron 1 of the Coagulation Factor IX (*F9*) gene.

## Material and methods

### Collection of study samples

The extended family P101 was described in great detail in the original linkage studies [2, 3, 15]. The family originates from a genetic isolate in Eastern Finland where the population only stabilized in the 17^th^ century, prior to a major expansion [16]. This was also one of the genetic hubs for the so-called Finnish disease heritage, which refers to a group of rare recessive disorders that due to genetic bottlenecks have become more prevalent in Finland than elsewhere in the world [17]. Pekkarinen et al. also alluded to BD being more enriched in this region compared to the nation in general [2].

Altogether, 24 out of 61 family members were diagnosed with a psychiatric disorder using the DSM-III-R (Diagnostic and Statistical Manual of Mental Disorder III-R) criteria. Out of these, 10 were diagnosed with BD (BD type I:8, BD type 2:1, BD not otherwise specified:1), one with schizoaffective disorder of bipolar type, five with recurrent major depression, two with schizophrenia, one with schizophreniform disorder, two with psychosis not otherwise specified, and three with alcohol abuse. The clinical DSM-IV diagnosis for this pedigree was last updated in 2002, when all the individuals (except two: 686 was 33 years-of-age, and 690 was 24 years-of-age) were aged 42 years or older, well beyond the mean age of onset for bipolar disorder which is 22 years.

The study to uncover the genetic etiology of bipolar disorder in family P101 was approved by the Helsinki university hospital’s epidemiology and public health ethics committee on June 11th, 2003 (Dnro189/E3/2003). Participants gave a written informed consent.

We selected 16 individuals from this X-chromosomally linked family for targeted SMRT sequencing, including carriers of the disease haplotype, non-carriers of the disease haplotype, and non-related controls that were married into the family. All DNA samples sequenced represented the third and fourth generation of the extended family and included five cases of bipolar type 1 and two cases of recurrent major depression cases (Fig 1).

### Targeted enrichment and sequencing

**Fig 2:**
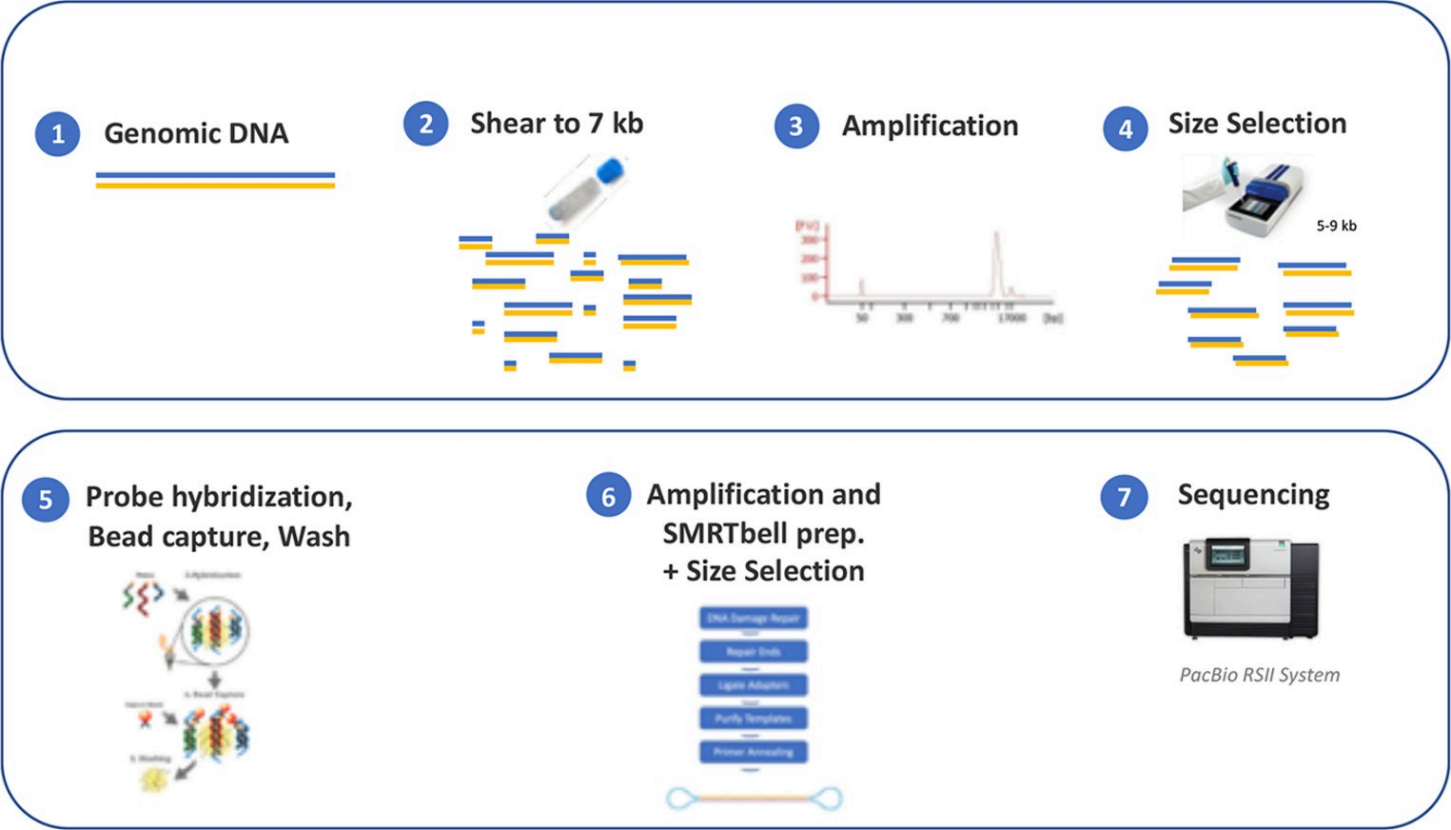
Overview of SeqCap^®^ EZ probe-based capture and subsequent SMRT sequencing workflow. Genomic DNA was sheared into on average of 10Kb fragments followed by a ligation of adapters, amplification and size selection step using the BluePippin System. Next, the probes where hybridized, followed by bead capture and washing to remove any nonspecific and unbound molecules. Finally, the sequencing libraries were generated, size selected using BluePippin and loaded onto the PacBio RS II sequencing system.

In order to survey the 12Mb significantly linked region, we used Roche Sequencing Solutions’ SeqCap EZ sequence capture technology in combination with SMRT^®^ sequencing. A custom designed SeqCap EZ probe pool (Hoffman-La Roche, Basel, Switzerland) was built tiling nearly 77% of the 12Mb region (GRCh38 ChrX:128,049,971–139,850,027). The capture experiment was performed in accordance with the protocol PN 100-893-500-0, available from Pacific Biosciences. Specifically, 2μg of genomic DNA from each of the 16 samples were sheared to an average of 10Kb fragments using g-Tube^TM^ by Covaris (PN 520079, Covaris, MA, USA). For each DNA sample, a library was made using the KAPA HyperPrep Kit^®^ for Illumina (Hoffman-La Roche). Libraries were amplified (12 cycles) under conditions optimized for long fragments before size selection using the BluePippin^®^ System (Sage Science, MA, USA) to a desired size ranged of 5-9Kb. Next, the SeqCap EZ probes were hybridized to 1.5μg of each DNA library, followed by bead capture and washing. The captured DNA fragments were then amplified (18 cycles) and PacBio SMRTbell^®^ sequencing libraries were prepared as recommended by the manufacturer. The final libraries were size selected on the BluePippin to a size of 6–6.5Kb. All size determinations were performed using a Fragment Analyzer (Agilent Technologies, CA, USA). The samples were not multiplexed during the library preparation process and were sequenced separately on individual SMRT Cells (3 for each sample, except for one sample with 2 SMRT Cells) on the PacBio RS II instrument (Fig 2).

### Sequencing analysis and visualization of results using Integrated Genome Viewer (IGV)

PacBio continuous long reads were mapped to the GRCh38 reference genome using pbmm2 v0.12.0 (https://github.com/PacificBiosciences/pbmm2), using the `—median-filter`flag to align one subread per Zero-Mode Waveguide (ZMW). Putative PCR duplicates were flagged with a custom script (https://github.com/williamrowell/markdup). The on-target rate was calculated as the proportion of ZMWs with a read overlapping the targeted region after deduplication. The results were visualized using IGV 2.5 [18] with Quick Consensus mode enabled and indels shorter than 3bp hidden.

### Structural variant detection

SVs were detected in the deduplicated alignments with the pbsv 2.0.1 workflow (https://github.com/PacificBiosciences/pbsv). The pbsv software will detect variants larger than 20bp. Using bedtools v2.27.1, we filtered for variants in the targeted region. Using bcftools v1.9, we filtered for variants with the requirement that an SV must be detected at least once in BD-affected individuals (685, 691, 698, 699, 700) and absent in the outgroup of non-X-chromosomal relatives (689, 690, 693, 697).

### Verification of the 330bp *Alu* element deletion using PCR and Sanger Sequencing

Forward and reverse primers (RBMX2_F: 5’-ACATTGCCAAATTGCTCTCC-3’ and RBMX2_R: 5’-CACCACCACACCTGGCTAC-3’) were designed to amplify the flanking region of *Alu* deletion in the intron 3 region of the *RBMX2* gene. To be able to amplify both the reference allele and the deletion allele within *RBMX2* (S1 Table), we designed two additional primers (RBMX2_Rref: 5’-CATATCTGACACCTTTAATTTCT**A**-3’ and RBMX2_Rdel: 5’-CATATCTGACACCTTTAATTTCT**G**). Reverse primers (RBMX2_Rref and RBMX2_Rdel) have a single nucleotide difference at the last 3’ position of the primer (A/G, shown in bold type here). This position is ChrX:130,406,896 in GRCh38 and also a known SNP (rs56113207). Two allele specific PCR reactions (RBMX2_F and RBMX2_Rref (1215bp, reference allele); RBMX2_F and RBMX2_Rdel (896bp, deletion allele) were performed for each of the 16 individuals.

All PCR reactions were performed in 50μl total reaction volume for each sample: DNA 1μl, 10x Buffer B1 5μl, MgCl_2_ (25mM) 3μl; dNTPs (10mM) 1μl; Primers (10μM) 2.5μl each; Phusion^®^ Hot Start II DNA Polymerase (2U/μl, ThermoFisher Scientific) or Hot FirePol Polymerase (5U/μl, Solis BioDyne, EE) 0.5μl; H_2_0 34.5μl. PCR reactions were performed on Veriti 96 well thermal cycler (ThermoFisher Scientific). Cycling conditions were as follows: polymerase activation 95°C for 15sec, then 35 amplification cycles of 95°C for 15sec, 63°C for 30sec and 72°C for 60sec with a final extension 72°C for 5min. PCR products were purified with AMPure^®^ Beads (Beckman Coulter, CA, USA).

The PCR product of RBMX2_F and RBMX2_Rdel (893bp) was sequenced using forward and reverse primers and Sanger sequencing. The longer reference-like i.e. sequence equivalent to current human genome sequence (Hg38), PCR product (1215bp) was sequenced using the forward and reverse primer (RBMX2_Rref). To resolve the obtained hairpin structure the longer reference-like PCR product was first cut with *ScaI* restriction enzyme and then sequenced with forward (RBMX2_F) and reverse (RBMX2_Rref) and two additional primers BRMX2_389 (5’-TCGATCTCTTGACCTCGTGA- 3’) and BRMX2_397 (5’-CGGATCACGAGGTCAAGAGA -3’). Finally, the original longer PCR product was cut with *BbvI* followed by sequencing with RBMX2_Long_BbvI_F (5’-TCATATCCTTTGCCAACTTTC-3’) primer. All Sanger sequencing used BigDye chemistry and was performed on Applied Biosystems 3130 Genetic Analyzer (ThermoFisher Scientific) for a small subset of family members (693, 688 and 700) that could serve as representatives for the other samples that showed the same variations based on PCR results and under the assumption that they would generate same Sanger sequencing results. To predict secondary structures of PCR amplified sequences we used Secondary Structure Web Server (http://rna.urmc.rochester.edu/RNAstructureWeb/Servers/Predict1/Predict1.html) with default structure options, selecting DNA was as a nucleic acid type.

### Sequence features and genome conservation analysis

Dfam (release 3.1, dfam.org/home) database was used to identify the family of *Alu* elements within intron 3 of *RBMX2* gene. Sequence homologies of the highlighted *Alu* element in intron 3 and the surrounding intron 3 sequence was explored using default parameters with the LAST [19] alignment tool and blastn [20]. In order to visualize genome conservation between species for the 330bp *Alu* element deletion in the *RBMX2* gene, the comparative genomics track in www.ensemble.org was utilized.

## Results

### Targeted enrichment using customized SeqCap EZ probe panel

**Fig 3:**
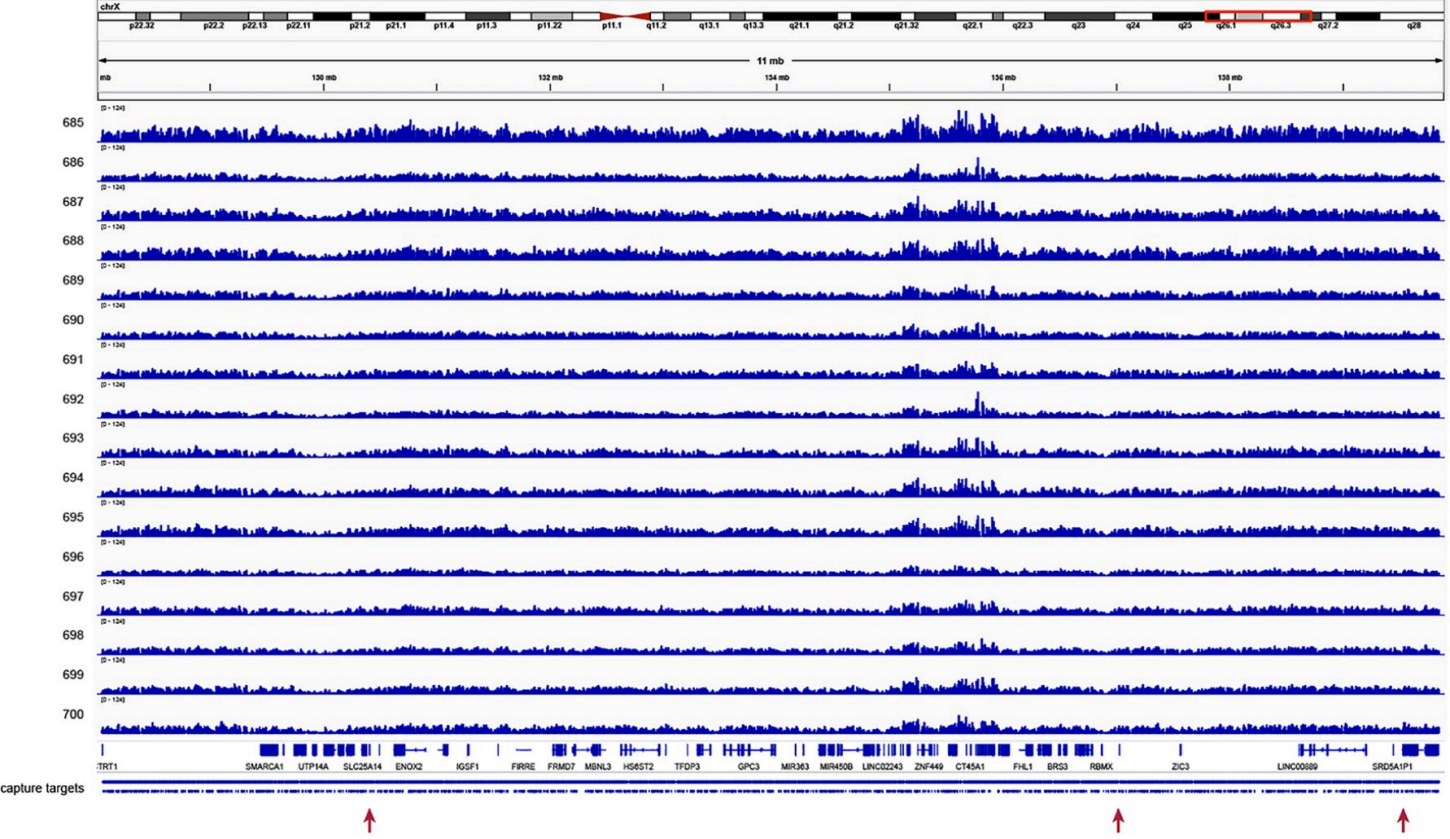
Sequencing coverage plot across the targeted 12Mb region on chromosome Xq24-q27 for all 16 family members. Each row represents the sequencing coverage for each family member separated by ID number. On the top, the chromosomal location is displayed and at the bottom, the genes and probe locations are shown. The red arrows indicate the locations of the three SVs that were highlighted in this study. The genes *RBMX2* (ChrX:130,405,824–130,406,154) and *F9* (ChrX:139,530,720–139,563,459) are not visible at this level of magnification.

SeqCap EZ capture efficiency ranged from 51% to 69%, with a mean of 61% on-target reads (S2 Table). Altogether 47 SMRT Cells were sequenced, with mean throughput of 572Mb per SMRT Cell. Unique molecular coverage of the targeted region ranged from 14.6-fold to 34.5-fold, with a mean of 21-fold sequencing coverage (Fig 3).

### Structural variation detection analysis

**Fig 4:**
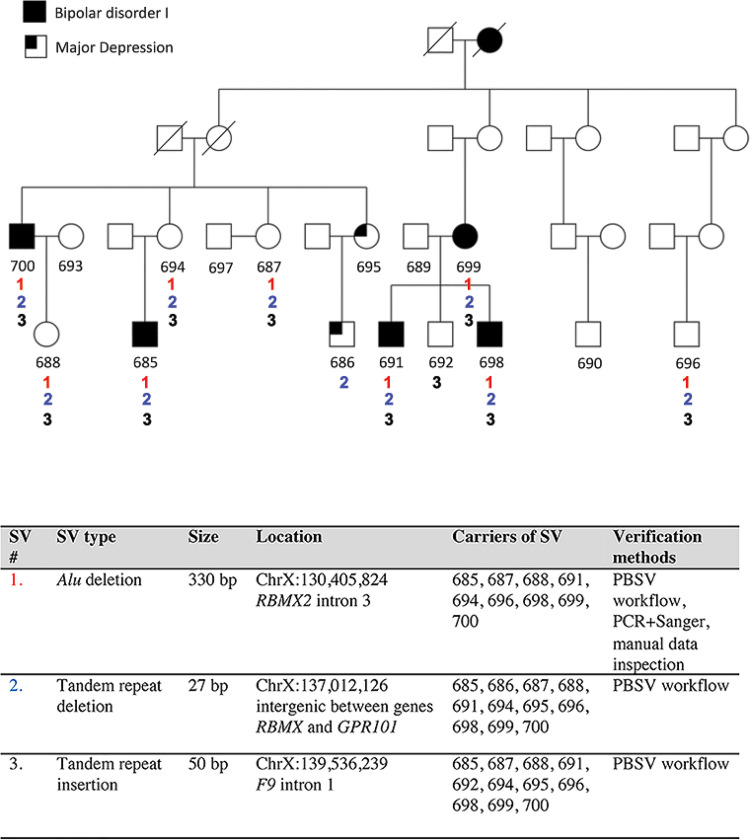
Candidate SVs. In total, three SVs were highlighted in this study. These included an *Alu* deletion (1, red), a tandem repeat deletion (2, blue) and a tandem repeat insertion (3, black) of various sizes. Two of them (1 and 2) were located in or in close vicinity of different *RBMX* gene family members, while one was located in the *F9* gene. The pedigree shows the segregation by number of each of the SVs.

Using the PBSV workflow, we identified 201 SVs within the 12Mb target region, however most represented common variants that could be seen across many of the family members, regardless of the disease status (S3 Table). From the set of variants seen in known carriers of the disease haplotype (685, 691, 698, 699, 700), we subtracted any variant seen in the haplotype non-carriers or married-in controls (689, 690, 693, 697), identifying 35 variants unique to carriers. Of these, only three variants were well-supported in four or more known carriers; a 330bp antisense *Alu* deletion in intron 3 of the *RBMX2* gene (ChrX:130,405,824–130,406,154), a 27bp tandem repeat deletion in the intergenic region between the *RBMX* and *GPR101* genes (ChrX:137,012,126–137,012,153) and a 50bp tandem repeat insertion in intron 1 of the *F9* gene (ChrX:139,536,239) (Fig 4).

### Verification of the *RBMX2* 330bp *Alu* element deletion using PCR and Sanger sequencing

We designed one forward and two allele specific reverse primers to amplify both reference-like and deletion alleles in region of *RBMX2*, intron 3. Based on PacBio reads, all 5 BPD-diagnosed individuals and four non-diagnosed but X-chromosomally linked individuals (687, 688, 694, 696) carried the C allele of this SNP while all others carried the T allele. Allele frequencies for this SNP in all populations tested are T: 84.3% and C: 15.7% and for the Finnish population T: 87.5% and C:12.5%, respectively (www.ensemble.org).

**Fig 5:**
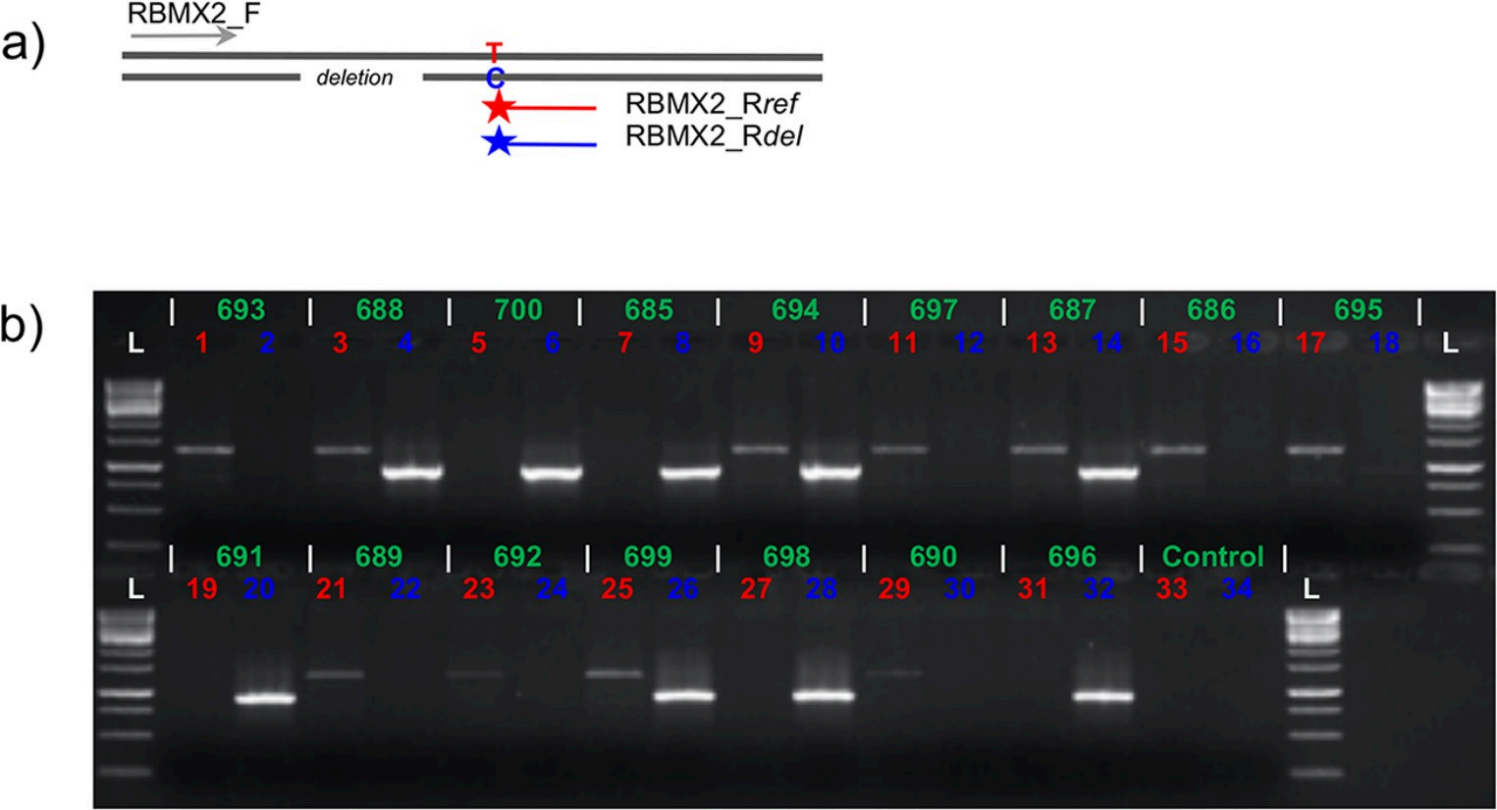
Verification of the *RBMX2* 330bp *Alu* deletion using PCR and Sanger Sequencing. (**a**) A schematic overview of the allele specific primer design. (**b**) An agarose gel image with two allele specific PCRs (a reference allele, 1215bp, and a 330bp deletion allele) were performed for each of the sixteen samples. Numbers colored with red are PCR reactions with RBMX2_F and RBMX2_Rref primers (a reference allele) and with blue RBMX2_F and RBMX2_Rdel (a deletion allele), respectively. **L** = 1kbp Ladder (ThermoFisher Scientific, CA, USA), **1–2**: female sample 693, **3–4**: female sample 688, **5–6**: male sample 700, **7–8**: male sample 685, **9–10**: female sample 694, **11–12**: male sample 697, **13–14**: female sample 687, **15–16**: male sample 686, **17–18**: female sample 695, **19–20:** male sample 691, **21–22:** male sample 689, **23–24:** male sample 692, **25–26:** female sample 699, **27–28:** male sample 698, **29–30:** male sample 690, **31–32:** male sample 696, **33–34:** Negative PCR controls. On the top row the sample ID numbers are listed in green with the corresponding well numbers on the agarose gel image. Below, the reference allele is marked in red and the deletion allele in blue.

We used PCR and Sanger sequencing to verify the *RBMX2* gene 330bp deletion. One forward and two allele specific reverse primers were designed to amplify the reference like region and the flanking region of the 330bp deletion (Fig 5A). DNA from 16 family members were PCR amplified with allele specific primer pairs RBMX2_F and RBMX2_Rref, RBMX2_F and RBMX2_Rdel (Fig 5B). Two different DNA polymerases, Phusion Hot Start II DNA Polymerase and HOT FIREPol^®^ DNA Polymerase, were tested. We observed that HOT FIREPol DNA Polymerase performed better, presumably due to a lack of 3’ → 5’ exonuclease activity.

Sanger sequencing was performed for a subset of the P101 family members (693, 688, and 700). The deletion allele (Fig 5B, wells 4 and 6) was sequenced using the RBMX2_F and RBMX2_Rdel primers. On our first attempt, we failed to sequence the longer PCR product of the reference allele (Fig 5B, wells 1 and 3). We hypothesize that the local sequence context generated a secondary hairpin structure due to consecutive opposite strand *Alu* elements after the denaturation step, therefore preventing elongation during Sanger sequencing. Subsequently, we used *ScaI* and *BbvI* restriction enzymes to cut the PCR product and disrupt hairpin formation before sequencing.

### Sequence features and genome conservation in the candidate *Alu* element

**Fig 6:**
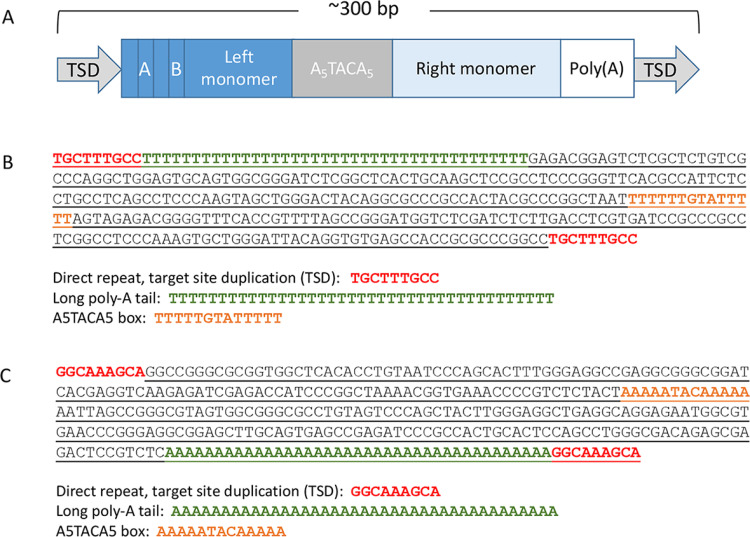
The *Alu* element sequence within *RBMX2* gene in intron 3. Direct repeats or target site duplications (TSDs) are shown in red, A_5_TACA_5_ box is shown in orange, and the long poly-A tail is shown in green. The 330 bp deleted sequence is underlined. (**a**) A structural scheme of an *Alu* element(21). (**b**) The sequence of the *Alu* element on the antisense strand (in relation to the *RBMX2* gene) in the GRCh38.p13 primary assembly (>NC_000023.11:130,405,825–130,406,163 Homo sapiens chromosome X). (**c**) The reverse complement sequence of the *Alu* element in the GRCh38.p13 primary assembly (>NC_000023.11:c130,406,163–130,405,825 Homo sapiens chromosome X).

**Fig 7:**
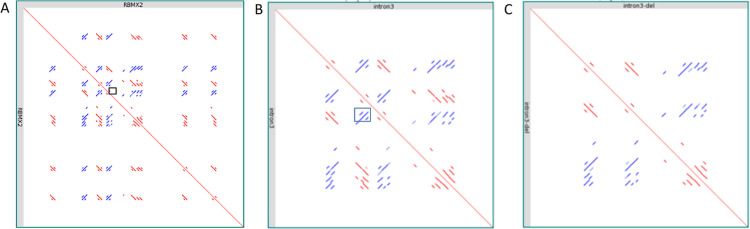
The sequence context of the *RBMX2* (11,688bp) gene. **(a**) The *RBMX2* gene sequence was aligned against itself. The red lines represent sequence homologies between sequences on the same strand, while blue lines represent sequence homologies on the antisense strand. The 330bp *Alu* element deletion in intron 3 is marked with a black box. (**b**) The intron 3 sequence is aligned in the *RBMX2* gene against itself. Again, the 330bp deletion is marked with a black box. (**c**) The 330bp deleted *Alu* element sequence is aligned against itself.

The highlighted *RBMX2 Alu* element deletion (ChrX:130,405,825–130,406,154) is on the antisense strand in relation to the *RBMX2* gene and it contains all sequence features characteristic to *Alu* elements [21] (Fig 6A). These consist of a nine bp (TGCTTTGCC) direct repeat and target site duplications (TDSs) that flank the *Alu* element. Also, in the middle, the *Alu* element contains a A_5_TACA_5_ -box as well as a 39 bp long A-tail on the 3’end (Fig 6B and 6C). After the 330bp deletion only one direct repeat remains of the *Alu* sequence. Based of Dfam database search, the *Alu* sequence is identified as *AluYa5* subfamily (e-value 2,2e-115). In order to explore how the 330bp *Alu* deletion may affect secondary structures several different sequence alignments were performed. First, the *RBMX2* gene (11.76Kb) and intron 3 (4.81Kb) sequences were aligned, both with and without the *Alu* deletion against itself (Fig 7A–7C). Most of the homologies seen are short but represent almost the full length of the *Alu* element like sequences.

**Fig 8:**
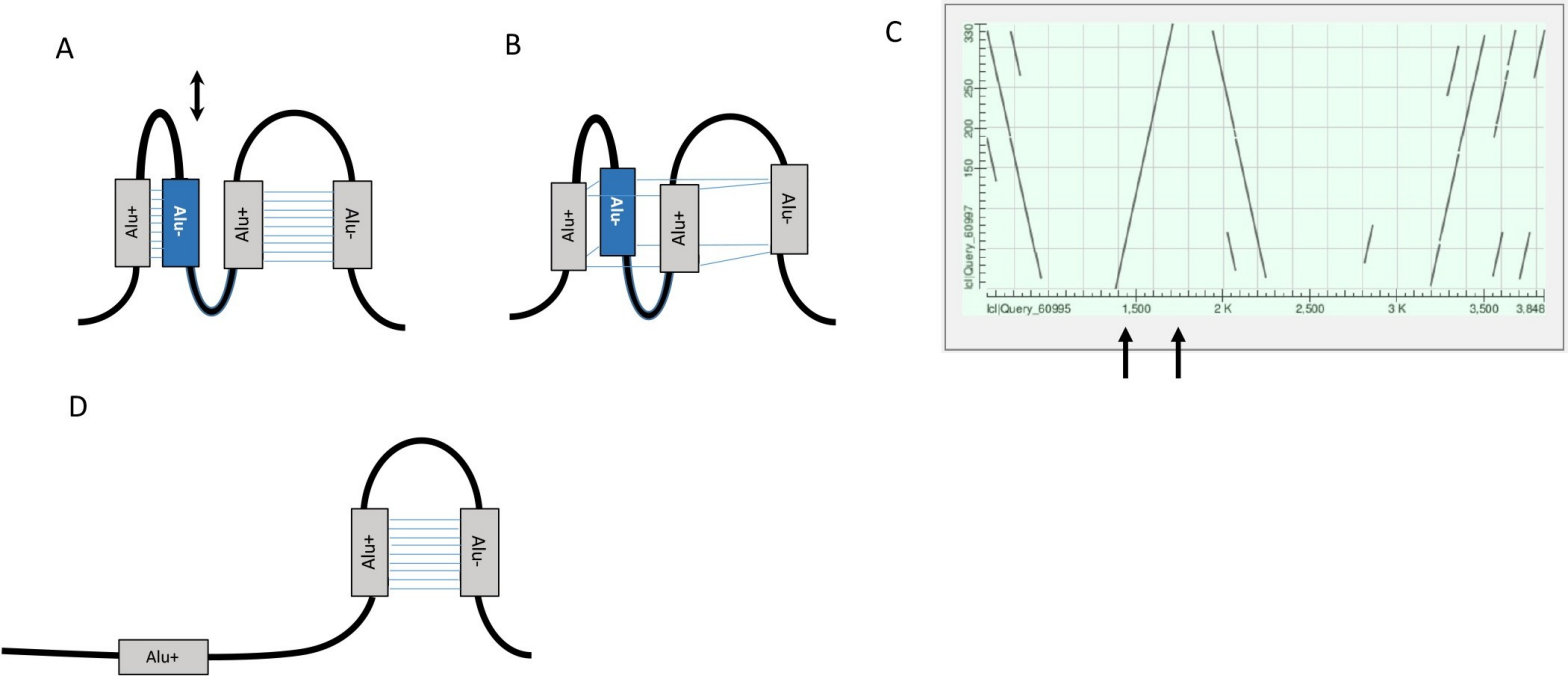
A schematic of the secondary structure of intron 3 in the *RBMX2* gene. Altogether four *Alu* elements can be found within this intron. The gray boxes represent reference like *Alu* elements while the blue *Alu* box represents the *Alu* element with the 330bp deletion. The sense and antisense strands are marked with a plus and minus sign, respectively. (**a**) The pre-mRNA secondary loops are shown for the *Alu* elements of opposite orientation. (**b**) A DNA *Alu* G-quadruplex structure is shown as described by Larsen et al 2018(22). (**c**) The full extent of the homologies between the *Alu* elements and the *RBMX2* gene intron 3 sequence are shown in this dotplot. The black arrows indicate the start and end positions of the *Alu* element with the 330bp deletion.

Altogether, four *Alu* elements, identified using Dfam, two (*AluSx* and *AluSx1)* on the sense and two (*AlueYa5* and *AluJr)* on the antisense strand, are found in intron 3 of the *RBMX2* gene (see Table 1*)*. The consecutive opposite *Alu* elements (*AluSx*, *AluYa5*, *AluSx1* and *AluJr*) with reference-like sequences would be expected to form hairpin loops in pre-mRNA as shown in Fig 8A, however at the DNA level, given the close proximity of the *Alu* elements, these may form a G quadruplex structure (Fig 8B). The homology between the four *Alu* elements can clearly be seen in Fig 8C, when comparing the sequence of the three reference-like *Alu* elements in intron 3 to the highlighted *Alu* element.

**Table 1:** *Alu* elements in the RBMX2 gene.

Sequence name	Model accession	Model name	Bit score	e-value	Model start	Model end	Strand	Alignment start	Alignment end	Envelope start	Envelope end
**Intron3**	**DF0000047**	**AluSx**	**340.6**	**1.6e-101**	**1**	**308**	**+**	**647**	**957**	**647**	**960**
Intron3	DF0000303	L1ME4a_3end	30.8	1.1e-07	725	869	-	1084	963	1096	962
Intron3	DF0000303	L1ME4a_3end	118.0	4.7e-34	160	504	-	1390	1099	1409	1088
**Intron3**	**DF0000053**	**AluYa5**	**382.8**	**3.1e-114**	**1**	**310**	**-**	**1704**	**1394**	**1704**	**1374**
Intron3	DF0000303	L1ME4a_3end	69.0	3.1e-19	1	172	-	1877	1708	1877	1705
**Intron3**	**DF0000048**	**AluSx1**	**320.7**	**1.8e-95**	**1**	**306**	**+**	**1942**	**2246**	**1942**	**2250**
Intron3	DF0000845	Tigger4a	28.8	2.2e-06	199	236	-	1947	1910	1954	1910
Intron3	DF0000846	Tigger4b	241.9	2.1e-71	1	341	-	2565	2243	2565	2240
Intron3	DF0000281	L1MC5a_3end	24.2	1.3e-06	1566	1694	-	2688	2567	2690	2546
Intron3	DF0000010	L1MC4_3end	46.4	2.2e-13	2679	2786	-	2812	2703	2834	2691
Intron3	DF0000147	FRAM	67.4	3.00E-18	66	168	-	2901	2799	2917	2791
Intron3	DF0000008	L1M5_orf2	20.0	1.5e-05	1915	2111	-	3061	2909	3087	2900
**Intron3**	**DF0000035**	**AluJr**	**247.8**	**2.5e-73**	**4**	**311**	**-**	**3506**	**3200**	**3509**	**3195**
Intron3	DF0001071	SVA_E	84.0	2.3e-24	133	317	+	3626	3835	3625	3855
Intron3	DF0000144	FLAM_C	106.5	3.2e-30	1	139	-	3680	3552	3680	3533
Intron3	DF0000144	FLAM_C	122.7	3.3e-35	1	139	-	3847	3709	3847	3689
Intron3	DF0000008	L1M5_orf2	120.3	9.1e-36	63	1042	-	4773	3848	4794	3846

The four different types of *Alu* elements (**bolded**) were identified in intron 3 of the *RBMX2* gene. Two were found on the sense strand and two on the antisense strand. Identification was done using Dfam.

**Fig 9:**
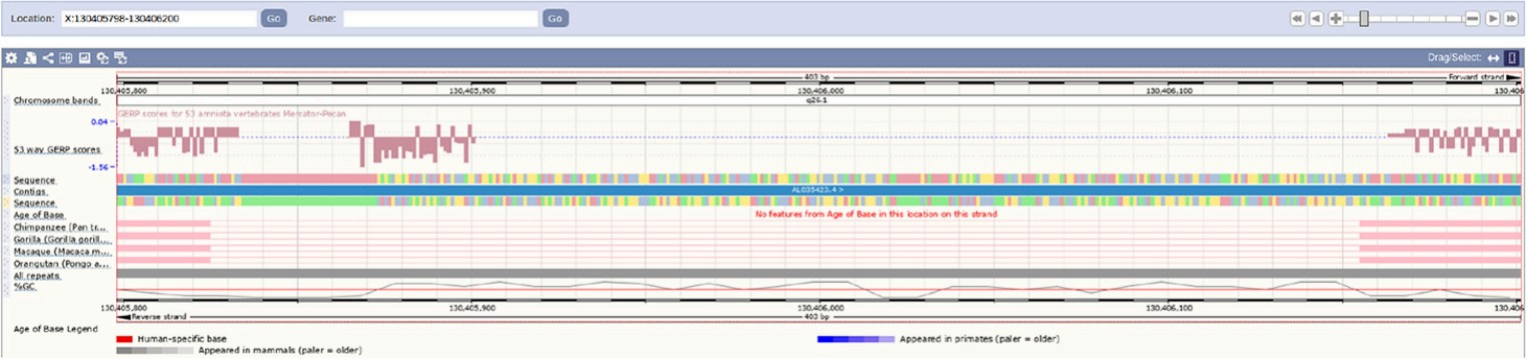
Cross-species evolutionary comparison of *Alu* deletion within intron 3 of the *RBMX2* gene (ChrX:130,405,798–130,406,200). On the top, the human sequence is compared to the following primates: chimpanzee, gorilla, macaque and orangutan. The target site duplications (TDSs: TGCTTTGCC) are indicated with black arrows flanking the *Alu* element and the polypyrimidine track is marked with a dashed black line.

To further explore the potential structural and functional relevance of this *Alu* element, the *Alu* sequence and flanking region was compared against the reference genomes of other primates, including chimpanzee, gorilla, macaque and orangutan. The 330bp *Alu* deletion allele was not observed in any of these non-human primates (Fig 9).

## Discussion

An extended pedigree segregating BD in four generations was originally found to be linked (lod score: 3.54) to Xq24-q27 in a genome-wide survey using microsatellite markers [2]. A follow-up study narrowed down the critically linked region to 12Mb [3]. In order to pinpoint disease associated variants, we used long-read sequencing to fully characterize the 12Mb genomic region in 16 out of the 61 family members. The P101 pedigree, which originates from a genetic isolate in Northern Finland, portrays a highly elevated recurrence risk for BD compared to the general population [1]. It has been hypothesized that the genetic heterogeneity would be reduced in isolated populations, even for genetically complex disorders such as BD [22]. This seems to also be the case for this extended family as it showed no evidence of significant linkage to any other chromosomal region in the original genome-wide scans [3], therefore this family may display a more monogenic form of the disorder.

In order to survey the critically linked 12Mb region, Roche’s hybridization-based target enrichment in combination with PacBio long-read sequencing was used. The SeqCap EZ probe pool was designed to enrich for 5-9Kb fragments spanning the entire 12Mb region. Compared to more traditional sequencing technologies, SMRT Sequencing has previously shown to be more efficient in capturing the full range of variations, including larger structural variations [10]. This technology has also demonstrated the ability to overcome other short-read sequencing barriers like accessing difficult to sequence regions such as long repeat expansions [23] with uniform and unbiased sequencing coverage [24]. The multi-kilobase sequence reads also help allelic phasing across large regions [25].

The SV analysis identified in total 201 SVs among the sixteen individuals in the 12Mb genomic region, however, the majority represented common variants seen across many family members regardless of disease status and therefore are not likely to be linked to BD. Nevertheless, three SVs emerged that showed verifiable segregation among the affected family members only.

Interestingly, two of the SVs were found in or near the *RBMX*-gene family; a 330bp antisense *Alu* element deletion in the intron 3 of the *RBMX2* gene and a 27bp tandem repeat deletion in the intergenic region between the *RBMX* and *GPR101* genes. The third SV was a 50bp tandem repeat insertion in the *F9* gene, which is a coagulation factor associated with HEMB (Hemophilia B) and THPH8 (Thrombophilia, X-Linked, due to factor IX defect). Although not an obvious candidate gene for the etiology of BD, the *F9* gene does encode for vitamin K- dependent coagulation Factor IX that circulates in the blood, and alterations of this gene, including point mutations, insertions and deletions, cause Factor IX deficiency. This is a recessive X-linked disorder, also called hemophilia B or Christmas disease that has shown linkage to both major depressive disorder as well as bipolar disorder in past studies [26–28].

The *RBMX* and *RBMX2* genes, located on Xq26, encode RNA-binding proteins that have several roles in the regulation of pre- and post-transcriptional processes [29, 30]. They have been implicated in tissue-specific regulation of gene transcription and alternative splicing of several pre-mRNAs that can either activate or suppress exon inclusion [29, 30]. Mutations in *RBMX* have also been associated with X-linked syndromic mental retardation-11(MRXS11; OMIM 300238). Shashi and colleagues identified a hemizygous 23bp deletion in exon 9 of the *RBMX* gene, resulting in a frameshift and premature termination [31, 32]. The mutation, which was found by whole-exome sequencing and confirmed by Sanger sequencing, segregated with the disorder in the family and was not found in the Exome Sequencing Project database or in 1300 controls. Here, we detected a 27bp tandem repeat deletion in the intergenic region between *RBMX* and the *GPR101* genes that segregated together in all the BD type I diagnosed P101 family members, as well as in one affected with major depression. In addition, four unaffected family members (687, 688, 694, 696) were also carriers of the tandem repeat variant. The *GPR101* gene is primarily expressed in brain and encodes a G protein-coupled receptor. G protein-coupled receptors are embedded in the outer membrane of cells, where they relay chemical signals from outside of the cell to the interior. The *GPR101* protein is predominantly expressed in the pituitary gland during fetal development and again at adolescence, two stages noted for rapid body growth [33, 34].

The *RBMX2* gene, located on Xq26.1 is over 11Kb in length and involved in pre-mRNA splicing. Here, we found a 330bp antisense *Alu* element deletion in intron 3 that again segregated in all P101 family members affected with BD type I, in addition to the same four unaffected family members (687, 688, 694, 696) as in the *RBMX* gene. All but 696 are females that might exhibit non-random X-inactivation where more of the mutant allele is expressed than in the unaffected female carriers in the family. The unaffected male 696 was 43 years old when initially entered into the study in 2002 and might have been pre-symptomatic with an expected later age of onset or simply a case of non-penetrance. The fact that the mean age of onset in this extended pedigree was 21 years would suggest the latter.

Like the *RBMX* gene, *RMBX2* has been shown to play a central role in brain development and function. ClinVar reported (submission accession #SCV000239951.1) on a SNP (rs5977266) in exon 6 that was characterized as a missense mutation and protein change (R287H) involved in abnormal neuronal migration. This finding falls within a diverse group of congenital brain developmental disorders that are characterized by defects in neuronal migration in the brain during early fetal development. These neuronal migration defects can result in brain abnormalities that are usually manifested with mental retardation and epilepsy [35]. The *RBMX2* gene is also located adjacent to the Solute Carrier Family 25 Member 14 (*SLC25A14*) gene, which has been shown to have altered expression in autism patients [36].

The highlighted 330bp antisense *Alu* element in the intron 3 of the *RBMX2* gene contained a direct repeat (TGCTTTGCC) flanking the element, a relatively long polypyrimidine tract, and a A_5_TACA_5_ box. *Alu* elements are known to be one of the most abundant repetitive elements in the human genome, with >1.3 million copies accounting cumulatively for at least 11% of human genomic DNA [22, 37]. They are approximately 300 nucleotides in length and tend to be accumulated in GC-rich regions that participate in the architecture of the genome by delimiting the active/inactive domains and the epigenetic landscape and gene regulation at different levels [38].

*Alu* elements are a highly successful family of primate-specific retrotransposons that have fundamentally shaped primate evolution, including human evolution [39]. The *Alus* play critical roles in the formation of neurological networks and the epigenetic regulation of biochemical processes throughout the central nervous system, and thus are hypothesized to have contributed to the origin of human cognition [39]. Despite the benefits that *Alu* elements may provide, deleterious *Alu* activity is associated with at least 37 neurological and neurodegenerative disorders, wherein *Alu* elements are hypothesized to disrupt key cellular processes that result in or contribute to the disease state [39]. *Alu* elements have many modes of action ranging from novel gene formation, elevated transcriptional diversity, long non-coding RNA and microRNA evolution (including circular RNAs), transcriptional regulation, and creation of novel response elements [40–44]. Moreover, *Alu* elements fundamentally alter the three-dimensional architecture and spatial organization of primate genomes by defining the boundaries of chromatin interaction domains (i.e., topologically associating domains (TADs) [45]. The deleted *Alu* element (subfamily *AluYa5)* in our current study is located on the antisense strand in relation to *RBMX2* gene. The *AluY* lineage is the youngest of the *Alu* lineages and has the largest number of functionally active sequences [46]. We observed that in the current human reference sequence (Hg38) the rather long (39 bp) stretch of homopolymeric adenosines (A-tail) we observed at the 3’ end of the *Alu* element here could indicate a more active *Alu* element [47].

It is known that polypyrimidine tracts within pre-messenger RNA (mRNA) promote the assembly of the spliceosome, the protein complex specialized for carrying out RNA splicing during the process of post-transcriptional modification [48]. Therefore, a deletion of a polypyrimidine tract may contribute to a change in 3’ splice site recognition. Also, the absence of polypyrimidine tract in pre-mRNA might have an impact on the polypyrimidine tract binding protein 1 (*PTB1*) involved in RNA looping when two separate pyrimidine tracts are present within the same RNA [49]. In fact, there are examples where mutations that alter polypyrimidine tract sequences may disrupt pre-mRNA splicing, leading to exon skipping or shortening, partial or full intron retention in the mRNA [50]. There is no specific known mechanism for removal of a complete *Alu* element [51], but the direct identical repeats flanking the *Alu* element are believed to be involved in recombination [50]. Here we found two direct repeats of 9 bp long (TGCTTTGCC) TSDs sequences flanking the intact *Alu* element allele whereas only one remained at the deletion allele. In addition to the complete *Alu* element in intron 3 of the *RBMX2* gene, we found three additional young *Alu* elements (*AluSx*, *AluSx1 and AluJr)* in the intron 3 region. The consecutive elements were in close proximity on opposite strands, which opens up the possibility of them forming pre-mRNA secondary loops or on the DNA-level, *Alu* G-quadruplex structures [39]. These non-B DNA *Alu* G-quadruplex structures are shown to serve as potential binding sites for proteins such p53, tumor suppressor protein, involved in complicated regulatory network by activating and repressing expression of ~1000 human genes [52]. In addition, Cui et al observed that the strongest binding sites for p53 reside in the relatively young *Alu* elements located in introns [52].

The *RBMX2* gene in itself is an important protein for the first step of splicing [53] which could further compromise transcription of candidate genes if compromised. In addition, we also found no evidence of the presence of this *Alu* element in other primate species, therefore making it specific to humans which would be expected if it was part of the mechanism leading to psychiatric disease.

In conclusion, targeted long read sequencing using probe-based hybrid-capture serves as a powerful tool to characterize the structure of regions linked to or associated with common complex disorders and may ultimately help pinpoint functional variants underlying the developmental or biochemical etiology of those disorders. In this study three SVs were highlighted, each an interesting candidate as a disease-causing variant for BD. However, the 330bp *Alu* deletion in the *RBMX2* gene emerged as the strongest candidate due to evidence supporting the hypothesis that loss of a complete *Alu* (subfamily *AluYa5*) element from within the *RBMX2* gene may be involved in the disease development of BD type I in this extended family. Here, we located two opposite strand *Alu-Alu* element pairs in intron 3 of this gene. This type of consecutive sense and antisense *Alu* element can potentially at pre-mRNA level form long double stranded RNA (dsRNA) structures and play a role in regulation of alternative splicing [54]. Due to this antisense 330bp Alu deletion, one of the *Alu*-*Alu* base-pairings cannot form the dsRNA, therefore this *Alu* deletion may influence the RNA splicing and generate novel transcripts in the RBMX2 gene. In order to fully understand the exact mechanism of how this 330bp deletion would be involved in the development of BD type I—further studies are warranted. For example, full-length isoform characterization and expression studies of the *RBMX2* gene would allow better delineation on how the downstream gene products are affected by this variant and how it could contribute to disease development. Additional, functional studies may also be warranted such as mouse models to fully elucidate the underlying disease mechanism.

## Supporting information

S1 Raw images Raw blot/gel image. The raw image for Fig 5.
(PDF)

S1 Table PCR experiment details. A description of wells, sample identification numbers and primer pairs used in the PCRs illustrated in the gel picture (Fig 5B)
(DOCX)

S2 Table Sequencing on-target rates. Sequencing summary for 16 family members. On-target rate is calculated as the number of unique templates for a given sample after deduplication.
(DOCX)

S3 Table Sequencing genotypes. Genotypes for all 16 family members at positions of the 35 variants identified as present in carriers and absent in non-blood related controls. Positions are provided in GRCh38 coordinates. In variant length column, positive sizes indicate insertions, and negative sizes indicate deletions. Genotypes are reported by PBSV as diploid, and encoded as 0 (reference allele), 1 (alternate allele), or (undetermined). Homozygous reference (0/0) or undetermined phenotypes (,/,) are shown in black, and homozygous alternate (1/1) or heterozygous (0/1) phenotypes are shown in red. Below the genotypes the number of supporting reads (REF, ALT) are shown in parentheses the total informative reads used by PBSV (which may not equal total overall coverage) are show in square brackets. The three highlighted variants are bolded and marked with an asterisk. The P101 family member affected with BD type I are underlined.
(DOCX)
