# Supplementary figures and images for: Alu element in the RNA binding motif protein, X-linked 2 (RBMX2) gene found to be linked to bipolar disorder
Source: PLoS One. 2021 Dec 16;16(12):e0261170. doi: 10.1371/journal.pone.0261170 (PMC8675739; doi:10.1371/journal.pone.0261170)

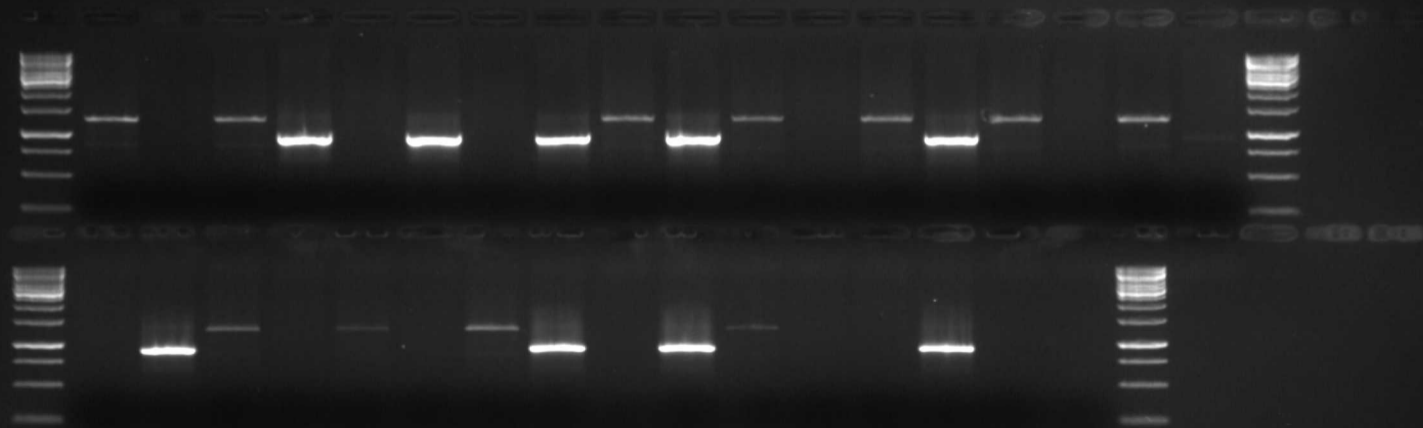

Supplement: S1 Raw images — The raw image for Fig 5. (PDF) [file pone.0261170.s001.pdf]
