# Supplementary material for: Alu element in the RNA binding motif protein, X-linked 2 (RBMX2) gene found to be linked to bipolar disorder
Source: PLoS One. 2021 Dec 16;16(12):e0261170. doi: 10.1371/journal.pone.0261170 (PMC8675739; doi:10.1371/journal.pone.0261170)
Supplement: S1 Table — A description of wells, sample identification numbers and primer pairs used in the PCRs illustrated in the gel picture (Fig 5B) (DOCX) [file pone.0261170.s002.docx]

**S1 Table 1.** **PCR experiment details**

| **Well** | **ID#** | **Forward primer** | **Reverse primer** |
| --- | --- | --- | --- |
| **L** | GeneRuler 1kb ladder |  |  |
| **1** | 693 | RBMX2_F | RBMX2_Rref |
| **2** | 693 |  | RBMX2_Rdel |
| **3** | 688 |  | RBMX2_Rref |
| **4** | 688 |  | RBMX2_Rdel |
| **5** | 700 |  | RBMX2_Rref |
| **6** | 700 |  | RBMX2_Rdel |
| **7** | 685 |  | RBMX2_Rref |
| **8** | 685 |  | RBMX2_Rdel |
| **9** | 694 |  | RBMX2_Rref |
| **10** | 694 |  | RBMX2_Rdel |
| **11** | 697 |  | RBMX2_Rref |
| **12** | 697 |  | RBMX2_Rdel |
| **13** | 687 |  | RBMX2_Rref |
| **14** | 687 |  | RBMX2_Rdel |
| **15** | 686 |  | RBMX2_Rref |
| **16** | 686 |  | RBMX2_Rdel |
| **17** | 695 |  | RBMX2_Rref |
| **18** | 695 |  | RBMX2_Rdel |
| **L** | GeneRuler 1kb ladder |  |  |
|  |  |  |  |
| **Well** | **ID** | **Forward primer** | **Reverse primer** |
| **L** | GeneRuler 1kb ladder |  |  |
| **19** | 691 | RBMX2_F | RBMX2_Rref |
| **20** | 691 |  | RBMX2_Rdel |
| **21** | 689 |  | RBMX2_Rref |
| **22** | 689 |  | RBMX2_Rdel |
| **23** | 692 |  | RBMX2_Rref |
| **24** | 692 |  | RBMX2_Rdel |
| **25** | 699 |  | RBMX2_Rref |
| **26** | 699 |  | RBMX2_Rdel |
| **27** | 698 |  | RBMX2_Rref |
| **28** | 698 |  | RBMX2_Rdel |
| **29** | 690 |  | RBMX2_Rref |
| **30** | 690 |  | RBMX2_Rdel |
| **31** | 696 |  | RBMX2_Rref |
| **32** | 696 |  | RBMX2_Rdel |
| **33** | Neg |  | RBMX2_Rref |
| **34** | Neg |  | RBMX2_Rdel |
| **L** | GeneRuler 1kb ladder |  |  |

A description of wells, sample identification numbers and primer pairs used in the PCRs illustrated in the gel picture (Fig 5b).
