# Supplementary material for: Alu element in the RNA binding motif protein, X-linked 2 (RBMX2) gene found to be linked to bipolar disorder
Source: PLoS One. 2021 Dec 16;16(12):e0261170. doi: 10.1371/journal.pone.0261170 (PMC8675739; doi:10.1371/journal.pone.0261170)
Supplement: S2 Table — Sequencing summary for 16 family members. On-target rate is calculated as the number of unique templates for a given sample after deduplication. (DOCX) [file pone.0261170.s003.docx]

**S2 Table. Sequencing on-target rates**

| ID# | On-target templates | Total templates | On-target rate |
| --- | --- | --- | --- |
| 685 | 77,078 | 115,570 | 66.7% |
| 686 | 47,979 | 93,042 | 51.6% |
| 687 | 65,557 | 98,574 | 66.5% |
| 688 | 65,291 | 95,025 | 68.7% |
| 689 | 52,088 | 91,207 | 57.1% |
| 690 | 48,406 | 84,249 | 57.5% |
| 691 | 54,781 | 95,012 | 57.7% |
| 692 | 45,849 | 75,867 | 60.4% |
| 693 | 52,342 | 80,762 | 64.8% |
| 694 | 52,651 | 81,492 | 64.6% |
| 695 | 55,105 | 84,333 | 65.3% |
| 696 | 35,650 | 69,920 | 51.0% |
| 697 | 47,123 | 83,737 | 56.3% |
| 698 | 44,213 | 79,302 | 55.8% |
| 699 | 49,045 | 71,608 | 68.5% |
| 700 | 50,193 | 89,387 | 56.2% |

Sequencing summary for 16 family members. On-target rate is calculated as the number of unique templates for a given sample after deduplication.
