# Supplementary material for: Alu element in the RNA binding motif protein, X-linked 2 (RBMX2) gene found to be linked to bipolar disorder
Source: PLoS One. 2021 Dec 16;16(12):e0261170. doi: 10.1371/journal.pone.0261170 (PMC8675739; doi:10.1371/journal.pone.0261170)
Supplement: S3 Table — Genotypes for all 16 family members at positions of the 35 variants identified as present in carriers and absent in non-blood related controls. Positions are provided in GRCh38 coordinates. In variant length column, positive sizes indicate insertions, and negative sizes indicate deletions. Genotypes are reported by PBSV as diploid, and encoded as 0 (reference allele), 1 (alternate allele), or (undetermined). Homozygous reference (0/0) or undetermined phenotypes (,/,) are shown in black, and homozygous alternate (1/1) or heterozygous (0/1) phenotypes are shown in red. Below the genotypes the number of supporting reads (REF, ALT) are shown in parentheses the total informative reads used by PBSV (which may not equal total overall coverage) are show in square brackets. The three highlighted variants are bolded and marked with an asterisk. The P101 family member affected with BD type I are underlined. (DOCX) [file pone.0261170.s004.docx]

**S3 Table. Sequencing genotypes**

| **Position** | **Variant ID** | **Variant Length** | **685** | **686** | **687** | **688** | **689** | **690** | **691** | **692** | **693** | **694** | **695** | **696** | **697** | **698** | **699** | **700** |
| --- | --- | --- | --- | --- | --- | --- | --- | --- | --- | --- | --- | --- | --- | --- | --- | --- | --- | --- |
| chrX:128,292,655 | pbsv.DEL.713 | -29 | 0/0 (5,0) [5] | 0/0 (8,0) [8] | 0/0 (6,0) [6] | 0/0 (6,0) [6] | 0/0 (5,0) [5] | 0/0 (6,0) [6] | 0/0 (4,0) [4] | 0/0 (12,0) [12] | 0/0 (7,0) [7] | 0/0 (7,0) [7] | 0/0 (2,0) [2] | 0/0 (10,0) [10] | 0/0 (7,0) [7] | 0/0 (5,0) [5] | 0/1 (6,2) [8] | 0/1 (10,1) [11] |
| chrX:129,431,404 | pbsv.INS.742 | 243 | ,/, (0,0) [0] | 0/0 (2,0) [2] | 0/0 (3,0) [3] | 0/1 (3,1) [4] | 0/0 (2,0) [2] | ,/, (0,0) [0] | 0/1 (0,1) [1] | 0/0 (2,0) [2] | 0/0 (1,0) [1] | 0/0 (1,0) [1] | 0/0 (1,0) [1] | 1/1 (0,2) [2] | 0/0 (1,0) [1] | 0/0 (1,0) [1] | 0/0 (2,0) [2] | 0/0 (2,0) [2] |
| chrX:129,904,295 | pbsv.DEL.743 | -26 | 0/0 (4,0) [4] | 0/0 (5,0) [5] | 0/0 (3,0) [3] | 0/0 (7,0) [7] | 0/0 (5,0) [5] | 0/0 (6,0) [6] | 0/0 (2,0) [2] | 0/1 (4,1) [5] | 0/0 (6,0) [6] | 0/0 (4,0) [4] | 0/1 (2,1) [3] | 0/0 (6,0) [6] | 0/0 (7,0) [7] | 0/0 (7,0) [7] | 0/0 (6,0) [6] | 0/1 (6,2) [8] |
| chrX:129,904,675 | pbsv.DEL.744 | -26 | 0/0 (5,0) [5] | 0/0 (7,0) [7] | 0/0 (5,0) [5] | 0/0 (7,0) [7] | 0/0 (5,0) [5] | 0/0 (6,0) [6] | 0/0 (2,0) [2] | 0/1 (4,1) [5] | 0/0 (4,0) [4] | 0/0 (5,0) [5] | 0/1 (1,1) [2] | 0/0 (6,0) [6] | 0/0 (8,0) [8] | 0/0 (7,0) [7] | 0/0 (5,0) [5] | 0/1 (7,2) [9] |
| chrX:129,928,397 | pbsv.DEL.745 | -22 | 0/1 (5,2) [7] | 0/0 (3,0) [3] | 0/1 (9,1) [10] | 0/0 (3,0) [3] | 0/0 (6,0) [6] | 0/0 (5,0) [5] | 0/0 (6,0) [6] | 0/1 (2,1) [3] | 0/0 (8,0) [8] | 0/1 (5,3) [8] | 0/0 (8,0) [8] | 0/0 (5,0) [5] | 0/0 (8,0) [8] | 0/0 (6,0) [6] | 0/0 (3,0) [3] | 0/0 (2,0) [2] |
| chrX:130,103,612 | pbsv.DEL.747 | -20 | 0/1 (13,1) [14] | 0/0 (6,0) [6] | 0/0 (14,0) [14] | 0/0 (14,0) [14] | 0/0 (11,0) [11] | 0/0 (14,0) [14] | 0/0 (6,0) [6] | 0/0 (6,0) [6] | 0/0 (5,0) [5] | 0/1 (12,1) [13] | 0/0 (15,0) [15] | 0/0 (6,0) [6] | 0/0 (13,0) [13] | 0/0 (8,0) [8] | 0/1 (7,2) [9] | 0/0 (7,0) [7] |
| chrX:130,288,182 | pbsv.DEL.748 | -21 | 0/1 (14,2) [16] | 0/1 (7,2) [9] | 0/0 (9,0) [9] | 0/1 (11,1) [12] | 0/0 (6,0) [6] | 0/0 (5,0) [5] | 0/0 (7,0) [7] | 0/0 (7,0) [7] | 0/0 (7,0) [7] | 0/1 (9,2) [11] | 0/0 (13,0) [13] | 0/1 (12,1) [13] | 0/0 (13,0) [13] | 0/0 (10,0) [10] | 0/0 (15,0) [15] | 0/0 (9,0) [9] |
| chrX:130,332,318 | pbsv.DEL.749 | -24 | 0/0 (14,0) [14] | 0/1 (10,1) [11] | 0/0 (12,0) [12] | 0/0 (9,0) [9] | 0/0 (6,0) [6] | 0/0 (6,0) [6] | 0/0 (9,0) [9] | 0/0 (9,0) [9] | 0/0 (2,0) [2] | 0/0 (11,0) [11] | 0/0 (9,0) [9] | 0/0 (11,0) [11] | 0/0 (11,0) [11] | 0/1 (8,1) [9] | 0/1 (13,1) [14] | 0/1 (7,2) [9] |
| **chrX:130,405,824** | **pbsv.DEL.751** | **-330*** | 1/1 (0,7) [7] | 0/0 (10,0) [10] | 0/1 (5,3) [8] | 0/1 (3,2) [5] | 0/0 (6,0) [6] | 0/0 (5,0) [5] | 0/1 (1,3) [4] | 0/0 (12,0) [12] | 0/0 (8,0) [8] | 0/1 (6,4) [10] | 0/0 (12,0) [12] | 1/1 (0,7) [7] | 0/0 (8,0) [8] | 1/1 (2,10) [12] | 0/0 (8,0) [8] | 0/1 (2,5) [7] |
| chrX:131,505,760 | pbsv.DEL.760 | -27 | 0/0 (16,0) [16] | 0/0 (8,0) [8] | 0/0 (8,0) [8] | 0/0 (11,0) [11] | 0/0 (9,0) [9] | 0/0 (9,0) [9] | 0/0 (10,0) [10] | 0/0 (15,0) [15] | 0/0 (6,0) [6] | 0/0 (4,0) [4] | 0/0 (5,0) [5] | 0/0 (7,0) [7] | 0/0 (10,0) [10] | 0/0 (7,0) [7] | 0/0 (10,0) [10] | 0/1 (6,2) [8] |
| chrX:131,871,444 | pbsv.DEL.763 | -3155 | ,/, (0,0) [0] | 0/0 (11,0) [11] | 0/1 (1,2) [3] | 0/1 (5,1) [6] | 0/0 (14,0) [14] | ,/, (0,0) [0] | ,/, (0,0) [0] | 0/0 (11,0) [11] | 0/0 (7,0) [7] | 0/1 (5,1) [6] | 0/0 (12,0) [12] | ,/, (0,0) [0] | ,/, (0,0) [0] | ,/, (0,0) [0] | 0/1 (4,1) [5] | ,/, (0,0) [0] |
| chrX:132,044,721 | pbsv.INS.784 | 68 | 0/0 (12,0) [12] | 1/1 (0,2) [2] | 0/0 (7,0) [7] | 0/0 (13,0) [13] | 0/0 (2,0) [2] | 0/0 (7,0) [7] | 0/0 (8,0) [8] | ,/, (0,0) [0] | 0/0 (11,0) [11] | 0/1 (4,1) [5] | ,/, (0,0) [0] | 0/0 (4,0) [4] | 0/0 (7,0) [7] | 0/0 (6,0) [6] | 0/1 (5,1) [6] | 0/0 (11,0) [11] |
| chrX:132,547,673 | pbsv.DEL.774 | -23 | 0/0 (12,0) [12] | 0/1 (9,1) [10] | 0/0 (15,0) [15] | 0/0 (8,0) [8] | 0/0 (12,0) [12] | 0/0 (8,0) [8] | 0/0 (10,0) [10] | 0/0 (10,0) [10] | 0/0 (15,0) [15] | 0/0 (8,0) [8] | 0/1 (14,2) [16] | 0/1 (9,1) [10] | 0/0 (12,0) [12] | 0/0 (8,0) [8] | 0/1 (6,1) [7] | 0/1 (5,3) [8] |
| chrX:132,718,627 | pbsv.DEL.776 | -23 | 0/1 (6,2) [8] | 0/0 (9,0) [9] | 0/0 (15,0) [15] | 0/0 (12,0) [12] | 0/0 (7,0) [7] | 0/0 (8,0) [8] | 0/0 (9,0) [9] | 0/0 (10,0) [10] | 0/0 (12,0) [12] | 0/0 (11,0) [11] | 0/0 (13,0) [13] | 0/0 (8,0) [8] | 0/0 (11,0) [11] | 0/0 (10,0) [10] | 0/0 (11,0) [11] | 0/0 (8,0) [8] |
| chrX:133,830,678 | pbsv.INS.798 | 23 | 0/0 (10,0) [10] | 0/1 (7,1) [8] | 0/0 (11,0) [11] | 0/0 (14,0) [14] | 0/0 (7,0) [7] | 0/0 (9,0) [9] | 0/0 (7,0) [7] | 0/0 (5,0) [5] | 0/0 (12,0) [12] | 0/1 (9,1) [10] | 0/1 (7,1) [8] | 0/0 (9,0) [9] | 0/0 (14,0) [14] | 0/0 (11,0) [11] | 0/1 (5,2) [7] | 0/1 (10,2) [12] |
| chrX:134,153,127 | pbsv.INS.815 | 37 | 0/1 (2,2) [4] | 0/0 (5,0) [5] | 0/0 (8,0) [8] | 0/0 (2,0) [2] | 0/0 (14,0) [14] | 0/0 (9,0) [9] | 0/0 (1,0) [1] | 0/0 (1,0) [1] | 0/0 (2,0) [2] | 0/0 (2,0) [2] | 0/0 (2,0) [2] | 0/0 (1,0) [1] | 0/0 (1,0) [1] | ,/, (0,0) [0] | 0/1 (2,1) [3] | 0/0 (1,0) [1] |
| chrX:134,195,083 | pbsv.INS.817 | 27 | 0/0 (2,0) [2] | 0/0 (3,0) [3] | 0/0 (5,0) [5] | 0/0 (2,0) [2] | 0/0 (4,0) [4] | 0/0 (3,0) [3] | 0/0 (5,0) [5] | 1/1 (0,3) [3] | 0/0 (6,0) [6] | 0/0 (3,0) [3] | 0/0 (5,0) [5] | 0/0 (4,0) [4] | 0/0 (7,0) [7] | 0/0 (2,0) [2] | 0/1 (3,2) [5] | ,/, (0,0) [0] |
| chrX:134,357,266 | pbsv.DEL.803 | -26 | ,/, (0,0) [0] | ,/, (0,0) [0] | ,/, (0,0) [0] | ,/, (0,0) [0] | ,/, (0,0) [0] | ,/, (0,0) [0] | ,/, (0,0) [0] | 0/1 (3,7) [10] | ,/, (0,0) [0] | ,/, (0,0) [0] | ,/, (0,0) [0] | ,/, (0,0) [0] | ,/, (0,0) [0] | ,/, (0,0) [0] | 0/1 (0,1) [1] | ,/, (0,0) [0] |
| chrX:134,492,000 | pbsv.DEL.807 | -34 | 0/0 (10,0) [10] | 0/0 (7,0) [7] | 0/0 (6,0) [6] | 0/0 (6,0) [6] | 0/0 (12,0) [12] | 0/0 (8,0) [8] | 0/0 (7,0) [7] | 1/1 (1,7) [8] | 0/0 (11,0) [11] | 0/0 (4,0) [4] | 0/0 (4,0) [4] | 0/0 (5,0) [5] | 0/0 (11,0) [11] | 0/0 (8,0) [8] | 1/1 (0,3) [3] | 0/0 (11,0) [11] |
| chrX:135,703,517 | pbsv.INS.833 | 52 | 0/0 (21,0) [21] | 0/0 (8,0) [8] | 0/0 (11,0) [11] | 0/0 (12,0) [12] | 0/0 (14,0) [14] | 0/0 (12,0) [12] | 0/0 (12,0) [12] | 0/0 (9,0) [9] | 0/0 (11,0) [11] | 0/0 (11,0) [11] | 0/0 (13,0) [13] | 0/0 (8,0) [8] | 0/0 (14,0) [14] | 0/0 (10,0) [10] | 0/1 (5,2) [7] | 0/0 (11,0) [11] |
| chrX:135,759,362 | pbsv.DEL.830 | -56 | ,/, (0,0) [0] | 1/1 (0,3) [3] | 0/1 (0,1) [1] | ,/, (0,0) [0] | ,/, (0,0) [0] | ,/, (0,0) [0] | ,/, (0,0) [0] | 0/1 (0,1) [1] | ,/, (0,0) [0] | 0/1 (0,1) [1] | 0/1 (0,1) [1] | ,/, (0,0) [0] | ,/, (0,0) [0] | ,/, (0,0) [0] | 0/1 (0,1) [1] | ,/, (0,0) [0] |
| chrX:135,924,892 | pbsv.INS.854 | 24 | 0/0 (17,0) [17] | 0/1 (14,1) [15] | 0/0 (7,0) [7] | 0/0 (17,0) [17] | 0/0 (8,0) [8] | 0/0 (8,0) [8] | 0/0 (8,0) [8] | 0/0 (11,0) [11] | 0/0 (8,0) [8] | 0/0 (12,0) [12] | 0/1 (6,2) [8] | 0/1 (6,1) [7] | 0/0 (14,0) [14] | 0/1 (12,1) [13] | 0/0 (8,0) [8] | 0/0 (12,0) [12] |
| chrX:136,278,563 | pbsv.DEL.843 | -329 | 0/1 (9,1) [10] | 0/0 (9,0) [9] | 0/0 (9,0) [9] | 0/0 (6,0) [6] | 0/0 (11,0) [11] | 0/0 (9,0) [9] | 0/0 (10,0) [10] | 0/0 (4,0) [4] | 0/0 (7,0) [7] | 0/1 (7,2) [9] | 0/0 (7,0) [7] | 0/0 (11,0) [11] | 0/0 (8,0) [8] | 0/0 (10,0) [10] | 0/0 (8,0) [8] | 0/1 (12,1) [13] |
| chrX:136,771,955 | pbsv.INS.859 | 21 | 0/1 (8,1) [9] | 0/0 (6,0) [6] | 0/0 (12,0) [12] | 0/0 (3,0) [3] | 0/0 (7,0) [7] | 0/0 (9,0) [9] | 0/0 (8,0) [8] | 0/0 (12,0) [12] | 0/0 (5,0) [5] | 0/0 (12,0) [12] | 0/0 (10,0) [10] | 0/0 (7,0) [7] | 0/0 (3,0) [3] | 0/1 (9,1) [10] | 0/0 (5,0) [5] | 0/1 (3,2) [5] |
| chrX:136,828,921 | pbsv.DEL.852 | -20 | 0/0 (11,0) [11] | 0/0 (6,0) [6] | 0/0 (8,0) [8] | 0/0 (10,0) [10] | 0/0 (9,0) [9] | 0/0 (6,0) [6] | 0/0 (10,0) [10] | 0/0 (7,0) [7] | 0/0 (8,0) [8] | 0/0 (10,0) [10] | 0/1 (8,1) [9] | 0/0 (7,0) [7] | 0/0 (9,0) [9] | 0/1 (7,2) [9] | 0/0 (6,0) [6] | 0/0 (9,0) [9] |
| chrX:136,878,636 | pbsv.INS.860 | 23 | 0/1 (11,1) [12] | 0/0 (7,0) [7] | 0/0 (9,0) [9] | 0/0 (11,0) [11] | 0/0 (10,0) [10] | 0/0 (7,0) [7] | 0/1 (5,3) [8] | 0/0 (8,0) [8] | 0/0 (7,0) [7] | 0/0 (6,0) [6] | 0/0 (11,0) [11] | 0/0 (6,0) [6] | 0/0 (6,0) [6] | 0/1 (5,2) [7] | 0/0 (7,0) [7] | 0/1 (3,1) [4] |
| **chrX:137,012,126** | **pbsv.DEL.863** | **-27*** | 0/1 (5,4) [9] | 1/1 (1,13) [14] | 0/1 (3,8) [11] | 0/1 (6,7) [13] | 0/0 (13,0) [13] | 0/0 (14,0) [14] | 0/1 (6,9) [15] | 0/0 (6,0) [6] | 0/0 (9,0) [9] | 1/1 (1,6) [7] | 0/1 (4,6) [10] | 0/1 (4,1) [5] | 0/0 (6,0) [6] | 0/1 (5,5) [10] | 0/0 (4,0) [4] | 1/1 (1,4) [5] |
| chrX:137,471,092 | pbsv.DEL.869 | -20 | 0/0 (8,0) [8] | 0/0 (9,0) [9] | 0/0 (13,0) [13] | 0/0 (13,0) [13] | 0/0 (10,0) [10] | 0/0 (4,0) [4] | 0/0 (8,0) [8] | 0/0 (9,0) [9] | 0/0 (9,0) [9] | 0/0 (13,0) [13] | 0/0 (9,0) [9] | 0/1 (9,1) [10] | 0/0 (9,0) [9] | 0/0 (14,0) [14] | 0/0 (9,0) [9] | 0/1 (7,2) [9] |
| chrX:137,604,017 | pbsv.INS.879 | 23 | 0/0 (4,0) [4] | 0/0 (4,0) [4] | 0/1 (5,2) [7] | 0/1 (1,1) [2] | 0/0 (8,0) [8] | 0/0 (6,0) [6] | 0/1 (3,1) [4] | 0/0 (8,0) [8] | 0/0 (3,0) [3] | 0/0 (5,0) [5] | 0/0 (2,0) [2] | 0/1 (1,1) [2] | 0/0 (4,0) [4] | 0/0 (4,0) [4] | 0/1 (2,1) [3] | 0/0 (4,0) [4] |
| chrX:137,802,461 | pbsv.INS.880 | 40 | 0/1 (16,2) [18] | 0/0 (21,0) [21] | 0/0 (11,0) [11] | 0/0 (8,0) [8] | 0/0 (22,0) [22] | 0/0 (17,0) [17] | 0/1 (14,1) [15] | 1/1 (1,9) [10] | 0/0 (7,0) [7] | 0/0 (9,0) [9] | 0/0 (14,0) [14] | 0/0 (13,0) [13] | 0/0 (14,0) [14] | 0/0 (10,0) [10] | 0/1 (12,5) [17] | 0/1 (12,2) [14] |
| chrX:138,549,061 | pbsv.DEL.888 | -23 | 0/0 (16,0) [16] | 0/1 (15,4) [19] | 0/0 (16,0) [16] | 0/1 (19,1) [20] | 0/0 (15,0) [15] | 0/0 (10,0) [10] | 0/0 (12,0) [12] | 0/0 (7,0) [7] | 0/0 (15,0) [15] | 0/0 (8,0) [8] | 0/0 (14,0) [14] | 0/1 (9,1) [10] | 0/0 (8,0) [8] | 0/1 (12,1) [13] | 0/1 (10,1) [11] | 0/0 (6,0) [6] |
| chrX:139,352,500 | pbsv.DEL.896 | -25 | 0/1 (11,1) [12] | 0/0 (8,0) [8] | 0/0 (10,0) [10] | 0/1 (3,1) [4] | 0/0 (13,0) [13] | 0/0 (6,0) [6] | 0/0 (9,0) [9] | 0/0 (5,0) [5] | 0/0 (2,0) [2] | 0/1 (7,2) [9] | 0/0 (10,0) [10] | 0/0 (8,0) [8] | 0/0 (12,0) [12] | 0/0 (7,0) [7] | 0/0 (11,0) [11] | 0/0 (8,0) [8] |
| chrX:139,503,636 | pbsv.INS.910 | 110 | 0/1 (9,2) [11] | 0/1 (4,1) [5] | 0/1 (7,4) [11] | 0/1 (12,6) [18] | 0/0 (5,0) [5] | 0/0 (6,0) [6] | 0/0 (6,0) [6] | 0/1 (5,1) [6] | 0/0 (7,0) [7] | 0/0 (5,0) [5] | 0/0 (8,0) [8] | 0/0 (4,0) [4] | 0/0 (8,0) [8] | 0/0 (8,0) [8] | 0/0 (4,0) [4] | 0/0 (1,0) [1] |
| **chrX:139,536,239** | **pbsv.INS.911** | **50*** | 1/1 (1,18) [19] | 0/0 (7,0) [7] | 0/1 (10,4) [14] | 0/1 (4,3) [7] | 0/0 (7,0) [7] | 0/0 (12,0) [12] | 1/1 (0,7) [7] | 1/1 (0,7) [7] | 0/0 (8,0) [8] | 0/1 (4,4) [8] | 0/1 (11,1) [12] | 1/1 (1,10) [11] | 0/0 (12,0) [12] | 1/1 (2,8) [10] | 1/1 (0,8) [8] | 0/1 (11,2) [13] |
| chrX:139,771,320 | pbsv.DEL.905 | -23 | 0/0 (5,0) [5] | 0/0 (3,0) [3] | 0/0 (4,0) [4] | 0/0 (4,0) [4] | 0/0 (5,0) [5] | 0/0 (5,0) [5] | 0/0 (4,0) [4] | 0/0 (3,0) [3] | 0/0 (6,0) [6] | 0/1 (2,2) [4] | 0/0 (2,0) [2] | 0/0 (3,0) [3] | 0/0 (6,0) [6] | 0/0 (2,0) [2] | 0/0 (2,0) [2] | 0/1 (8,1) [9] |

Genotypes for all 16 family members at positions of the 35 variants identified as present in carriers and absent in non-blood related controls. Positions are provided in GRCh38 coordinates. In variant length column, positive sizes indicate insertions, and negative sizes indicate deletions. Genotypes are reported by PBSV as diploid, and encoded as 0 (reference allele), 1 (alternate allele), or (undetermined). Homozygous reference (0/0) or undetermined phenotypes (,/,) are shown in black, and homozygous alternate (1/1) or heterozygous (0/1) phenotypes are shown in red. Below the genotypes the number of supporting reads (REF, ALT) are shown in parentheses the total informative reads used by PBSV (which may not equal total overall coverage) are show in square brackets. The three highlighted variants are bolded and marked with an asterisk. The P101 family member affected with BD type I are underlined.
